# Sex Disparities in *MGMT* Promoter Methylation and Survival in Glioblastoma: Further Evidence from Clinical Cohorts

**DOI:** 10.3390/jcm10040556

**Published:** 2021-02-03

**Authors:** Anja Smits, Malgorzata Lysiak, Andreas Magnusson, Johan Rosell, Peter Söderkvist, Annika Malmström

**Affiliations:** 1 Department of Neuroscience and Physiology, Clinical Neuroscience, Sahlgrenska Academy, Gothenburg University, Blå Stråket 7, Plan 3, SE-413 45 Gothenburg, Sweden; 2 Department of Neuroscience, Neurology, Uppsala University Hospital, SE-751 85 Uppsala, Sweden; 3 Department of Biomedical and Clinical Sciences, Linköping University, SE-581 85 Linköping, Sweden; malgorzata.lysiak@liu.se (M.L.); peter.soderkvist@liu.se (P.S.); annika.malmstrom@regionostergotland.se (A.M.); 4 NU-Hospital, SE-451 53 Uddevalla, Sweden; magnusson.andreas@me.com; 5 Regional Cancer Center South East Sweden, Region Östergötland, SE-581 85 Linköping, Sweden; johan.rosell@regionostergotland.se; 6 Clinical Genomics Linköping, Science for Life Laboratory, Linköping University, SE-581 85 Linköping, Sweden; 7 Department of Advanced Home Care, Linköping University, SE-581 85 Linköping, Sweden

**Keywords:** glioblastoma, *MGMT* promoter methylation, survival, sex disparities

## Abstract

Introduction: Recent studies suggest an overrepresentation of *MGMT* promoter methylated tumors in females with *IDH*wt glioblastoma (GBM) compared to males, with a subsequent better response to alkylating treatment. Methods: To reveal sex-bound associations that may have gone unnoticed in the original analysis, we re-analyzed two previously published clinical cohorts. One was the multicenter Nordic trial of elderly patients with GBM, randomizing patients into three different treatment arms, including 203 cases with known *MGMT* promoter methylation status. The other was a population-based study of 179 patients with *IDH*wt GBM, receiving concomittant radiotherapy and chemotherapy with temozolomide. Cohorts were stratified by sex to test the hypothesis that female sex in combination with *MGMT* promoter methylation constitutes a subgroup with more favorable outcome. Results: There was a significantly larger proportion of *MGMT* promoter methylation and better outcome for female patients with *MGMT* promoter methylated tumors. Results were confirmed in 257 TCGA-derived *IDH*wt GBM with known sex and *MGMT* status. Conclusions: These results confirm that patient sex in combination with *MGMT* promoter methylation is a key determinant in GBM to be considered prior to treatment decisions. Our study also illustrates the need for stratification to identify such sex-bound associations.

## 1. Introduction

Glioblastoma (GBM) is the most common malignant primary brain tumor in adults, occurring mostly in the fifth and sixth decade of life [1]. The incidence of GBM is around 3.2 per 100,000 inhabitants [2]. First line treatment consists of maximal safe surgery, followed by radiotherapy (RT) and chemotherapy [3]. In spite of multimodal strategies, the median survival of patients with *IDH*wt GBM is less than 18 months [2,4]. 

Similar to many other cancers, GBM occurs more frequently in the male population (male-to-female ratio 1.6:1) [5,6]. The potential influence of patient sex on the disease is intriguing and has received increased attention [7,8,9]. Of particular interest in this context are sex-bound disparities in the epigenetic regulation of glioma, and how they impact downstream gene expression. There is accumulating evidence that gliomas display sex-specific methylation patterns [10,11]. *MGMT* promoter methylation is more commonly found in females with GBM, with a subsequent better outcome after treatment with alkylating agent temozolomide (TMZ) [12,13,14,15]. Interestingly, this prognostic advantage for females is not present in gliomas of lower malignancy grade [16]. Since *MGMT* promoter methylation is the most powerful biomarker for response to alkylating agent treatment in GBM at present, these findings have important implications for tailored therapy and need confirmation by further studies [17].

To address this issue, we re-analyzed the datasets from two previously published GBM cohorts. Patient sex as a parameter was included in the original survival analyses, but no sub-analysis searching for specific interactions with regard to sex was made at that time. Based on the assumption that sex-bound associations may have gone unnoticed, we hypothesized that re-evaluation of data after stratification by sex may unravel differences in *MGMT* promoter methylation between female and male patients [18]. For confirmation of findings, a cohort of *IDH*wt GBM derived from The Cancer Genome Atlas (TCGA) was used.

## 2. Materials and Methods

### 2.1. Patient Cohorts

The first cohort was the NORDIC multicenter randomized study of elderly patients (60 years or older) with GBM diagnosis obtained by biopsy or tumor resection, and enrolled into a three-arm trial, comparing standard RT (60 Gy) with hypofractionated RT (34 Gy) or TMZ [19]. A total of 342 patients were included and randomized between three different treatment arms (temozolomide *n* = 119; hypofractionated RT *n* = 123, standard RT *n* = 100). *MGMT* methylation status, determined by methylation specific PCR, could be assessed for 203 patients, this being 59% of the total study cohort (Table 1). No follow-up was performed after publication of the study.

The second cohort was a population-based cohort of 179 patients from South-East Sweden with *IDH*wt GBM and known *MGMT* methylation status, analyzed by pyrosequencing (Table 1). Patients were recruited from 2004 and onwards and followed until death or until last follow-up (1 December 2020) [20]. Histological diagnosis of *IDH*wt GBM was obtained by biopsy or tumor resection, and all patients received postoperative RT concomitant with TMZ.

As a third, confirmatory cohort, we searched TCGA for *IDH*wt GBM with known sex and *MGMT* methylation status, determined by bead-based microarray technology. We identified 257 patients (106 females, 151 males) of whom 189 received alkylating agent therapy, either with TMZ or a nitrosourea compound (Table 1).

### 2.2. Statistical Analyses

To test for sex-bound differences in the proportion of tumors with methylated *MGMT* promoter (m*MGMT*) versus (vs.) unmethylated *MGMT* promoter (u*MGMT*), the Pearson’s Chi^2^ test was used. Type I error was set at 5% and all tests were two-tailed. Overall survival was estimated by Kaplan–Meier method with a two-sided Log-rank test. For comparison of hazard ratios (HR) for relative risk of death, multivariate Cox regression was used. 

## 3. Results

Table 1 shows the number of patients in the three cohorts and the parameters used for the present study.

**Figure 1:**
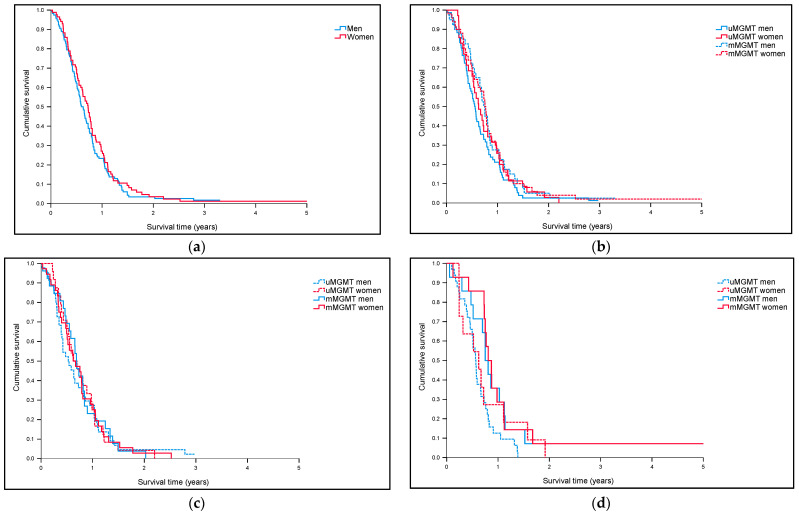
Overall survival in the NORDIC-trial (**a**) for men (*n* = 203) vs. women (*n* = 139) in the whole cohort (*p* = 0.37). (**b**) For men (*n* = 117) vs. women (*n* = 86) according to *MGMT* status (*p* = 0.40). (**c**) For men (*n* = 70) vs. women (*n* = 61) in the RT-arms (*p* = 1.0). (**d**) For men (*n* = 47) vs. women (*n* = 25) in the TMZ-arm of the trial (*p* = 0.056).

First Cohort (NORDIC trial): As shown in Table 1, the proportion of tumors with m*MGMT* was higher in females than in males (59% vs. 34%, *p* = 0.0004, Chi^2^ test). Median overall survival (MOS) for men was 7.0 months (CI 6.1–7.9) vs. 7.5 months (CI 6.4–8.6) for women in the whole cohort. In the subgroup of 203 patients with known *MGMT* status, MOS for men was 7.6 months (CI 6.5–8.6) vs. 8.7 months (CI 7.2–10.2) for women. There were no statistically significant differences in survival between the sexes in the whole cohort (Figure 1a) or according to *MGMT* status (Figure 1b).

In a next step, we performed separate analyses for patients included in the RT arms (standard RT or hypofractionated RT) (*n* = 131) or the TMZ-arm (*n* = 72) of the trial. As expected, no sex-bound differences according to *MGMT* status were seen for patients included in the RT-arms (u*MGMT* men = 44 vs. u*MGMT* women = 24 vs. m*MGMT* men = 26 vs. m*MGMT* women = 37) (*p* = 1.0, Log-rank) (Figure 1c). Figure 1d, on the other hand, shows differences in survival between men and women included in the TMZ-arm (u*MGMT* men = 33 vs. u*MGMT* women = 11 vs. m*MGMT* men = 14 vs. m*MGMT* women = 14). As illustrated, MOS was longest for women (9.7 months, CI 7.5–11.9) and men (9.0 months, CI 7.4–10.6) with m*MGMT*, compared to women (7.5 months, CI 2.9–12.0) and men (6.8 months (CI 5.8–7.9) with u*MGMT*, although numbers were small and differences did not reach statistical significance (*p* = 0.056, Log-rank) (Figure 1d).

We used sex-specific multivariate Cox regression in the TMZ-arm (70 males, 47 with known *MGMT* status; 49 females, 25 with known *MGMT* status), to test the impact of surgery (biopsy vs. resection), performance status (PS) (WHO 0–1 vs. WHO 2–3), *MGMT* status (u*MGMT* vs. m*MGMT*) and dichotomized age on survival in males respectively females. Table S1 shows the results. In females, tumor resection was associated with longer survival (*p* = 0.017). In males, m*MGMT* was associated with longer survival (*p* = 0.013), together with PS WHO 0–1 (<0.0001).

**Figure 2:**
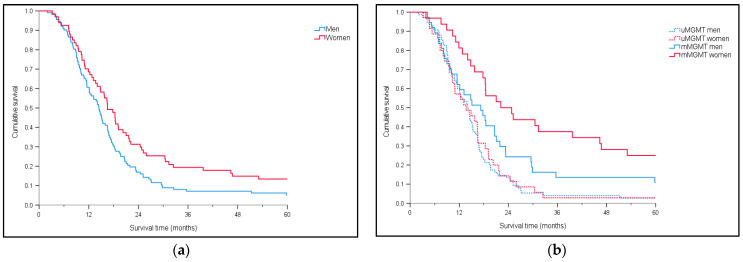
Overall survival in the population-based cohort (**a**) for men (*n* = 112) vs. women (*n* = 67) (*p* = 0.035). (**b**) For men (*uMGMT* = 75; *mMGMT* = 37) vs. women (*uMGMT* = 35; *mMGMT* = 32) according to *MGMT* status (*p* = 0.0002).

Second (population-based) cohort: As shown in Table 1, the proportion of *mMGMT* tumors was significantly higher in females compared to males (48% vs. 33%, *p* = 0.05, Chi-2 test) in the population-based cohort. The MOS in this cohort was 15.1 months (CI 13.5–16.8). Figure 2a shows a significantly shorter MOS for men (*n* = 112) than for women (*n* = 67) (*p* = 0.035, Log-rank). Figure 2b shows survival according to *MGMT* status (*p* = 0.0002, Log-rank), with longest survival for females with m*MGMT* (MOS females with *mMGMT* 22.1 months, CI 12.7–31.5; MOS males with *mMGMT* 17.3 months, CI 11.1–23.4). As expected, patients with u*MGMT* had poorest outcome (females with *uMGMT* 13.7 months, CI 8.1–19.2; males with *uMGMT* 14.1 months, CI 11.9–16.3).

Table S2 shows the results of sex-specific multivariate survival analysis in cohort 2, with the variables surgery (biopsy vs. partial resection vs. radical resection), PS (WHO 0–1 vs. WHO 2–3), *MGMT* status (u*MGMT* vs. m*MGMT*) and dichotomized age entered in the Cox regression model. In females, m*MGMT* was associated with longer survival (*p* = 0.0014), together with radical resection. In males, m*MGMT* was associated with longer survival (*p* = 0.024).

Third (TCGA-derived) cohort*:* Finally, we studied the correlation between *MGMT* status and sex in *IDH*wt GBM generated by TCGA Research Network: https://www.cancer.gov/tcga. A total of 151 males (55 m*MGMT*, 96 u*MGMT*) and 106 females (60 m*MGMT*, 46 u*MGMT*) were identified (Table 1). Consistent with reported findings, the proportion of m*MGMT* was significantly higher for women than for men (57% vs. 36%) (*p* = 0.001, Chi^2^ test). The MOS in this cohort was 12.9 months. Females with m*MGMT* had significantly longer MOS (17.2 months, CI 13.3–21.0) than females with u*MGMT* (11.8 months, CI 7.5–16.1), men with m*MGMT* (12.7 months, CI 8.6–16.8), and men with u*MGMT* (12.6 months, CI 11.1–14.1) (*p* = 0.01, Log-rank test; Figure S1a). Of the group of patients with m*MGMT* and treated with alkylating chemotherapy, either alone or in combination with RT, females had a statistically significant survival advantage (females 20.7 months, CI 19.3–30.0; males 15.8 months, CI 12.4–18.6) (*p* = 0.004, Log-rank) (Figure S1b).

## 4. Discussion

Sex-bound differences in susceptibility and survival of different cancer types are among the most consistent findings in cancer epidemiology that can be pivotal for developing a tailored therapy to cancer [7]. We tested the hypothesis that sex-associated disparities in *MGMT* promoter methylation may have gone unnoticed in the original analysis of two previously published clinical cohorts. For this purpose, we re-analyzed the datasets from the NORDIC trial of elderly GBM patients, and a population-based cohort of *IDH*wt GBM in our region receiving RT and chemotherapy. We found that approximately half of all women in both cohorts harbored m*MGMT*, while for men this proportion was around one third. These findings were robust and further confirmed by data from TCGA.

On top of sex-associated disparities in the proportion of m*MGMT*, there was a survival advantage for females in the population-based cohort, with longest survival in the subgroup of females with m*MGMT*. The results from TCGA database confirmed these data and showed a statically significant longer survival for females with m*MGMT* after alkylating agent treatment. For the NORDIC trial, no such survival differences were noted between men and women in the arm receiving TMZ treatment. This discrepancy was probably due to the low numbers (only 25 females with known *MGMT* status), reducing statistical power. Otherwise, sex-specific multivariate analysis confirmed m*MGMT* as a favorable prognostic factor in males (47 with known *MGMT* status) included in the TMZ-arm, and in males and females in the population-based cohort. Of the other established prognostic factors for GBM, tumor resection was associated with significantly longer survival for females in both cohorts. These findings give support for a favorable role of tumor resection on outcome also in the elderly population of GBM [21], and suggest that this benefit may not be similar for male and female patients. However, it should be noted that this is a preliminary observation that needs confirmation by larger studies. Also, different arrays to determine the *MGMT* promoter methylation status were used in the three cohorts, which may have affected the results.

Insight in the differences in epigenetic profiles between male and female patients will be vital for understanding the sex-bound discrepancies in gliomagenesis and prognosis, and may lead to improved treatments for both sexes. The study by Johansen and co-workers, exploring sex-specific gene methylation patterns in 587 glioma samples derived from TCGA, reported that the genes associated with hypermethylation in males with *IDH*wt GBM were enriched for cell cycle phase transition genes. In females, on the other hand, an enrichment of transcriptional regulators was seen, in agreement with the overrepresentation of m*MGMT* in females with GBM. Interestingly, methylation of the *MGMT* promoter does not seem to occur uniformly in a sex-bound fashion in all cancer types. A meta-analysis of the role of *MGMT* promoter methylation in small cell lung cancer showed a correlation with the clinical stage of this cancer type, but not with factors like sex, age, and smoking [22]. This suggests that the higher proportion of m*MGMT* and the better response to alkylating treatment seen in females with GBM is a tumor-specific phenomenon.

Our study confirms previous findings and exemplifies the need for stratification of the cohort by sex to unravel sex-bound differences that may otherwise go unnoticed. As pointed out by Dorak and Karpuzoglu [23], the concern over losing statistical power after stratification is not justified. On the contrary, by not splitting the sample into males and females there is a risk for not picking up gender-specific associations.

## 5. Conclusions

Taken together, we provide further evidence for sex-bound disparities in the epigenetic regulation of *IDH*wt GBM, exemplified by the *MGMT* status of the tumor, which could contribute to a survival advantage for female patients. Our data illustrate the need for stratification by sex in clinical cohorts of GBM, where an unbalanced incidence of the disease between males and females may disguise gender-specific associations with survival.

## Figures and Tables

**Table 1:** Clinical parameters of patients included in the three different cohorts.

	Total	Male	Female	*p*-Value
**1st cohort** (NORDIC trial)	342	203	139	
Known MGMT status	203	117	86	
Methylated *MGMT* promoter	91	40	51	0.0004
Unmethylated *MGMT* promoter	112	77	35	
**2nd cohort** (population-based)	179	112	67	
Methylated *MGMT* promoter	69	37	32	0.05
Unmethylated *MGMT* promoter	110	75	35	
**3rd cohort** (TCGA-derived)	257	151	106	
Patients treated with alkylating agent	189	116	73	
Methylated *MGMT* promoter	87	43	44	0.001
Unmethylated *MGMT* promoter	102	73	29	

## Supplementary Materials

The following are available online at https://www.mdpi.com/2077-0383/10/4/556/s1, Table S1: Multivariate Cox-regression in females and males in the TMZ-arm of cohort 1. Table S2: Multivariate Cox-regression in females and males in cohort 2. Figure S1a: Survival for men (*n* = 151) and women (*n* = 106) in 257 TCGA-derived *IDH*wt GBM with known *MGMT* status and sex (*p* = 0.01, Log-rank). Figure S1b: Survival for men (*n* = 116) and women (*n* = 73) in 189 TCGA-derived *IDH*wt GBM with known *MGMT* status and sex, treated with alkylating therapy (*p* = 0.004, Log-rank).
