# Supplementary material for: Sex Disparities in MGMT Promoter Methylation and Survival in Glioblastoma: Further Evidence from Clinical Cohorts
Source: J Clin Med. 2021 Feb 3;10(4):556. doi: 10.3390/jcm10040556 (PMC7913151; doi:10.3390/jcm10040556)

Table 1. Multivariate Cox-regression analysis of patients included in the TMZ-arm of cohort 1 (NORDIC trial).

MALES (*n*=70).

	Number of patients	Number of events	HR, 95% CI	P-value
Surgery				
Biopsy	19	19	1.00	-
Resection	51	49	0.78, 0.42-1.43	0.42
WHO				
0-1	54	52	1.00	-
2-3	16	16	4.01, 2.07-7.76	<0.0001
MGMT				
uMGMT	33	33	1.00	-
mMGMT	14	13	0.43, 0.22-0.83	0.013
Non valid/missing	23	23	0.51, 0.27-0.96	0.037
Age (median)				
≤70 years	39	38	1.00	-
>70 years	31	30	0.73, 0.44-1.21	0.22

FEMALES (*n*=49)

	Number of patients	Number of events	HR, 95% CI	P-value
Surgery				
Biopsy	14	14	1.00	-
Resection	35	34	0.42, 0.20-0.85	0.017
WHO				
0-1	37	36	1.00	-
2-3	12	12	1.77, 0.78-4.02	0.17
MGMT				
uMGMT	11	11	1.00	-
mMGMT	14	13	0.55, 0.24-1.30	0.17
Non valid/missing	24	24	0.98, 0.42-2.28	0.97
Age (median)				
≤71 years	28	27	1.00	-
>71 years	21	21	1.17, 0.60-2.26	0.65

Table S2. Multivariate Cox-regression analysis of patients included in the population-based cohort (cohort 2).

MALES (n=112)

	Number of patients	Number of events	HR, 95% CI	P-value
Surgery				
Biopsy	16	16	1.00	-
Partial surgery	25	24	1.05, 0.54-2.02	0.89
Radical surgery	70	65	0.75, 0.42-1.33	0.33
Non valid/missing	1	1	0.51, 0.07-3.85	0.51
WHO				
0-1	90	84	1.00	-
2-3	18	18	0.91, 0.54-1.53	0.71

Non valid/missing	4	4	2.01, 0.72-5.61	0.18
MGMT				
uMGMT	75	73	1.00	-
mMGMT	37	33	0.61, 0.40-0.94	0.024
Age (median)				
≤58 years	59	57	1.00	-
>58 years	53	49	1.37, 0.91-2.06	0.13

FEMALES (n=67)

	**Number of patients**	**Number of events**	**HR, 95% CI**	**P-value**
Surgery				
Biopsy	8	8	1.00	-
Partial surgery	17	17	0.67, 0.27-1.65	0.38
Radical surgery	40	36	0.33, 0.13-0.84	0.020
Non valid/missing	2	2	0.20, 0.03-1.25	0.085
WHO				
0-1	43	40	1.00	-
2-3	22	11	0.69, 0.37-1.29	0.25
Non valid/missing	2	2	3.02, 0.54-16.83	0.21
MGMT				
uMGMT	35	34	1.00	-
mMGMT	32	29	0.39, 0.22-0.70	0.0014
Age (median)				
≤59 years	35	33	1.00	-
>59 years	32	30	1.58, 0.90-2.76	0.11

Scheme S1a. Overall survival for men (n=151) and women (n=106) in 257 TCGA-derived primary GBM with known *MGMT* status and sex (p=0.01, Log-rank).

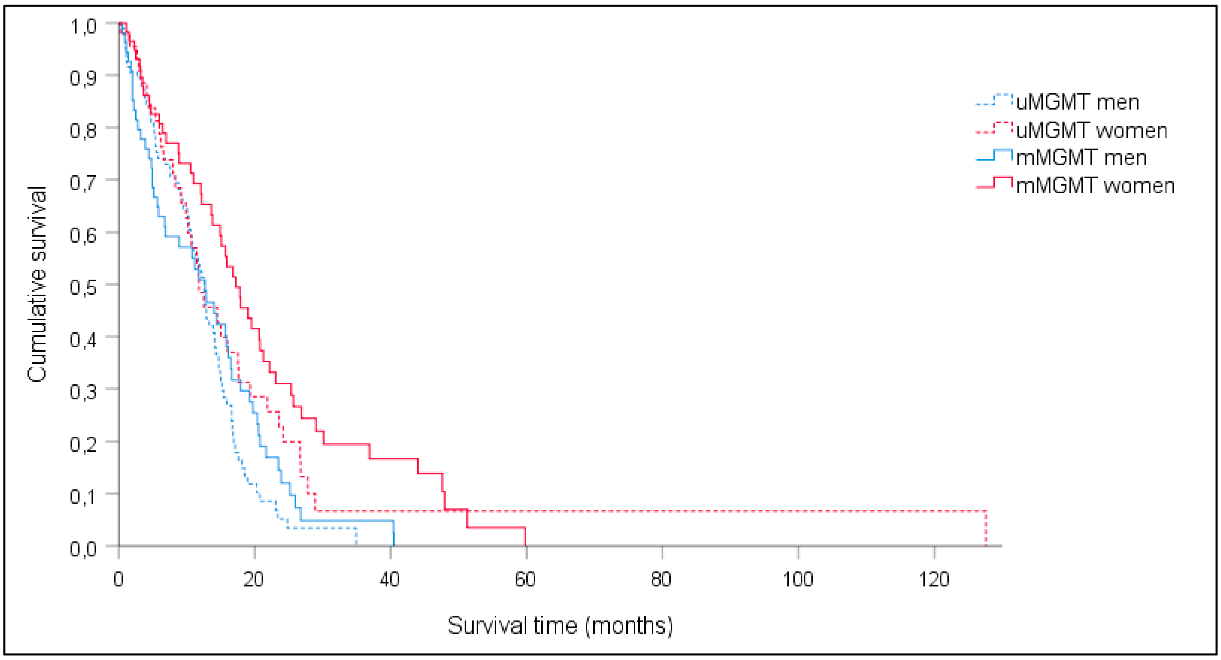

Scheme S1b. Overall survival for men (n=116) and women (n=73) in 189 TCGA-derived primary GBM with known *MGMT* status and sex, treated with alkylating therapy (p=0.004, Log-rank).

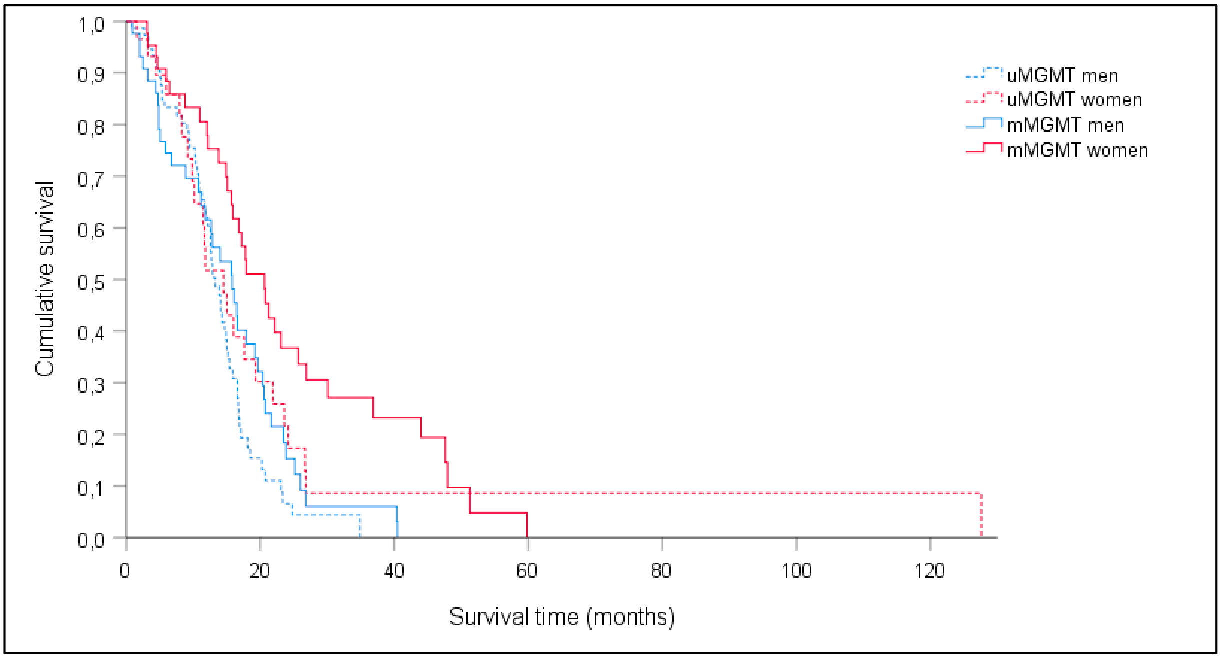

Supplement: Supplementary file 1 [file jcm-10-00556-s001.pdf]
